# Malignant Pheochromocytoma With Intravascular Extension to the Heart

**DOI:** 10.31486/toj.23.0049

**Published:** 2023

**Authors:** Salar Haider, Muhammad Ahmad Khan, Asif Islam, Rida E Fatima Mirza, Saman Aslam

**Affiliations:** ^1^ Department of Internal Medicine, Mayo Hospital, Lahore, Punjab, Pakistan; ^2^ Department of Internal Medicine, Jinnah Postgraduate Medical Centre, Karachi, Sindh, Pakistan

**Keywords:** *Adrenal glands*, *esophageal and gastric varices*, *metanephrine*, *pheochromocytoma*

## Abstract

**Background:** Malignant pheochromocytomas are rare and aggressive tumors that arise from the adrenal medulla and secrete catecholamines. Patients exhibit episodic symptoms of hypertension, headaches, sweating, and palpitations. The diagnosis is supported by elevated levels of urinary metanephrines, and imaging is used to determine the stage. Treatment involves surgical resection when possible.

**Case Report:** A 57-year-old male presented with hematemesis and melena, and endoscopy revealed upper gastrointestinal bleeding. Imaging showed a malignant pheochromocytoma that had infiltrated the upper lobe of the right kidney and the right lobe of the liver, with a tumor thrombus extending into the hepatic inferior vena cava, the right atrium, and the right ventricle. The patient denied surgery and was treated with palliative medical therapy until he died 3 months later.

**Conclusion:** Although rare, malignant pheochromocytomas may present with upper gastrointestinal bleeding. While metastasis to the liver is a typical manifestation of malignant pheochromocytomas, invasion of the inferior vena cava with infiltration to the right ventricle resulting in tricuspid valve malfunction is a rare finding.

## INTRODUCTION

Pheochromocytomas are tumors of neuroendocrine cells that secrete catecholamines.^1^ They originate most commonly in the adrenal gland medulla from chromaffin cells. Pheochromocytomas outside the adrenals are referred to as paragangliomas, and the majority arise from the sympathetic ganglia in the organ of Zuckerkandl.^2^

A 2022 systematic review reported that the incidence of these tumors ranges from 0.04 to 0.95 cases per 100,000 per year.^3^ While the majority of pheochromocytomas are sporadic and occur in patients in their forties, approximately 25% of pheochromocytomas are familial and exist as a part of hereditary neoplastic syndromes such as von Hippel-Lindau, neurofibromatosis type 1, and multiple endocrine neoplasia type 2 that are associated with germline mutations.^4-6^

Patients with pheochromocytomas present with paroxysmal symptoms of hypertension, headache, sweating, palpitations, dizziness, and anxiety.^7^ High levels of urinary or plasma metanephrines support the diagnosis with a test sensitivity of 98%.^8^ The tumor is localized as intra-adrenal or extra-adrenal, and possible metastasis is evaluated using computed tomography (CT) and magnetic resonance imaging. An immunohistochemical analysis of the tumor, usually done postoperatively, shows nests of chromaffin cells that stain positive from chromogranin.^9^

Approximately 13% to 23% of pheochromocytomas are malignant.^10^ Malignant tumors spread frequently to the regional lymph nodes, and among the larger organs, the liver is the most common site of metastasis.^11^ Malignant pheochromocytomas have also been reported to infiltrate the inferior vena cava (IVC) and cause variable levels of occlusion.^12,13^ The definitive treatment of pheochromocytomas is surgical resection; Rötker et al demonstrated that surgery can increase patient lifespan.^13^

We describe the case of a 57-year-old male who presented with upper gastrointestinal bleeding, and the diagnostic investigations identified an atypical manifestation of malignant pheochromocytoma.

## CASE REPORT

**Figure 1.**
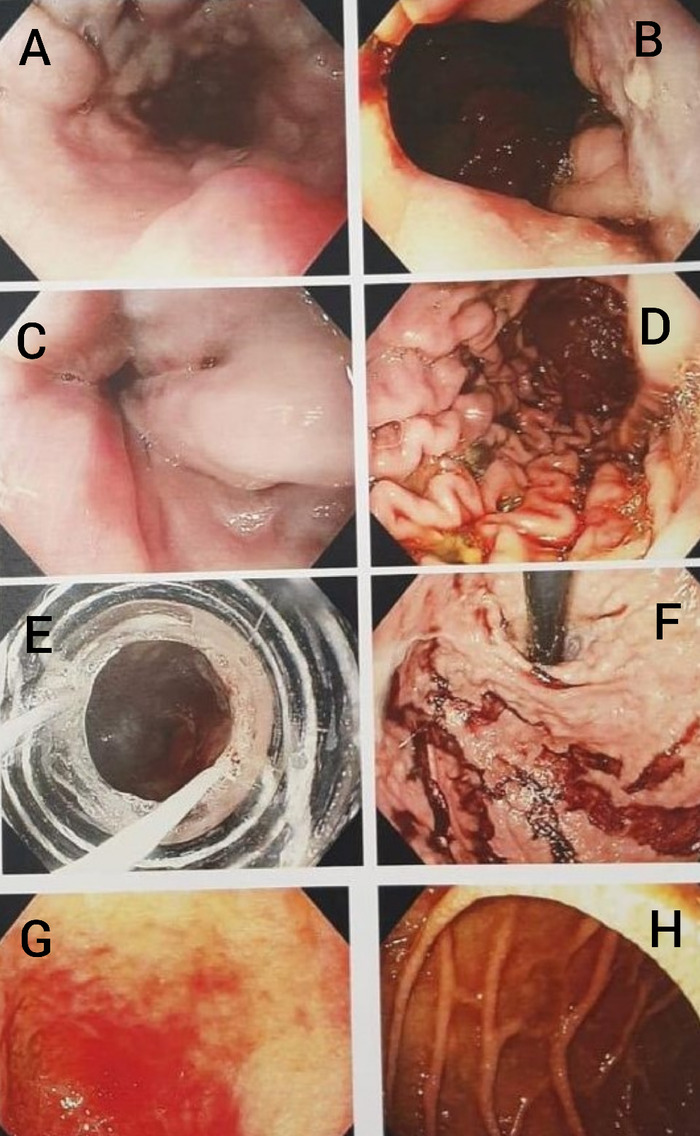
(A) Endoscopic view of the middle third of the esophagus shows grade 3 esophageal varices. (B) The gastroesophageal junction with view of the proximal stomach and distal esophagus shows high-grade varices. (C) Distal esophagus shows high-grade esophageal varices with red signs and an approximated lower esophageal sphincter. (D) Endoscopic view of the body of the stomach shows old blood and features of recent bleed from the varices. (E) View of the stomach through an endoscopic band ligator. (F) View of the fundus of the stomach and clotted blood. (G) Duodenal cap has features of duodenopathy. (H) Descending duodenum shows congested mucosa.

A 57-year-old male presented to the emergency and resuscitation unit with 3 episodes of hematemesis and melena since the prior day. Initial measures included administration of intravenous fluids and intravenous octreotide. After a brief and targeted examination revealing mild ascites, the patient underwent an emergency upper gastrointestinal endoscopy (Figure 1) that showed 3 columns of grade 3 lower esophageal varices with red signs and an ulcer oozing blood over the varix at the gastroesophageal junction. Blood clots were seen in the stomach, and no fundal varices were noted on retroflexion. An emergency variceal band ligation performed during the endoscopy successfully controlled the bleeding.

Once the patient was vitally stable, a detailed history was taken during which the patient revealed that he had had dull pain in his right flank for the prior few months that was associated with intermittent sweating, palpitations, and dizziness on standing. He also had an undocumented weight loss during this period and reported passing black-colored stools occasionally.

The patient was admitted to the inpatient ward and stayed for 2 weeks. During further workup for diagnosis and etiology, ultrasonography revealed a suprarenal mass adjoining the right lobe of the liver, along with coarse parenchymal changes in the liver and mild ascites. Tests for hepatitis B and C were negative and the patient denied any alcohol use in his lifetime, all of which are common causes of portal hypertension in our geographic region. In view of the patient's episodic symptoms and the adrenal mass detected on ultrasound, further investigations revealed plasma metanephrine of 1,140.6 pg/mL (reference range, 0 to 90 pg/mL), plasma normetanephrine of 32,260 pg/mL (reference range, 0 to 190 pg/mL), and urinary vanillylmandelic acid of 55 mg/24 hrs (reference range, <13.6 mg/24 hrs).

**Figure 2.**
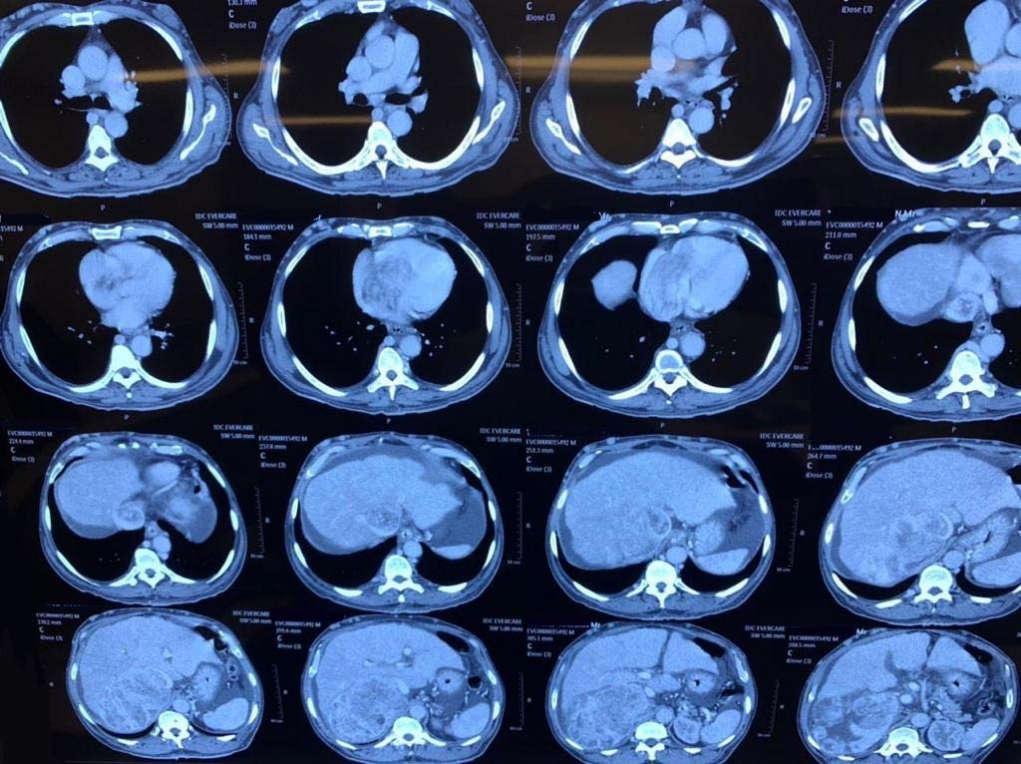
Computed tomography scan of chest and abdomen shows a heterogeneously enhancing mass in the right adrenal area with areas of low density suggesting necrosis. The mass measures 15 × 12 × 17 cm, is inseparable from the upper pole of the right kidney, and adjoins the right lobe of the liver (images in the third and fourth rows). Images in the second row show a partially occlusive thrombus in the inferior vena cava with extension of the thrombus into the right atrium and partially into the right ventricle. Secondary collaterals are seen developing in the chest and abdomen.

CT scan of the abdomen and chest with contrast showed a large necrotic mass centered at the right adrenal medulla infiltrating the upper lobe of the right kidney and the right lobe of the liver, with a tumor thrombus extending into the hepatic IVC, the right atrium, and the right ventricle (Figure 2). Extension into the right side of the heart was surprising because such a finding is rare.

**Figure 3.**
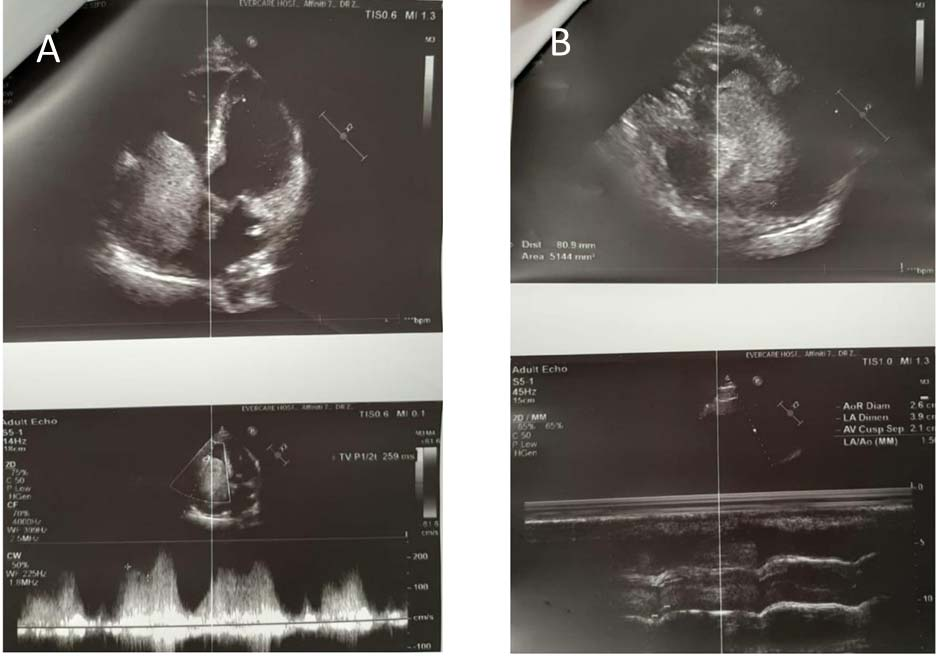
Echocardiology images show (A) the right atrium dilated with a mass measuring 85 × 50 mm, extending into the middle of the right ventricle through the tricuspid valve, and fully obstructing the valve. The tricuspid valve area is 0.75 cm^2^ by pressure half-time*.* (B) The left atrium is slightly dilated. The left ventricle and aortic root are normal size. Mitral valve leaflets show normal mobility with no prolapse or regurgitation.

Echocardiography confirmed a mass measuring 85 × 50 mm in the right atrium and the right ventricle that was causing tricuspid valve obstruction (Figure 3).

**Figure 4.**
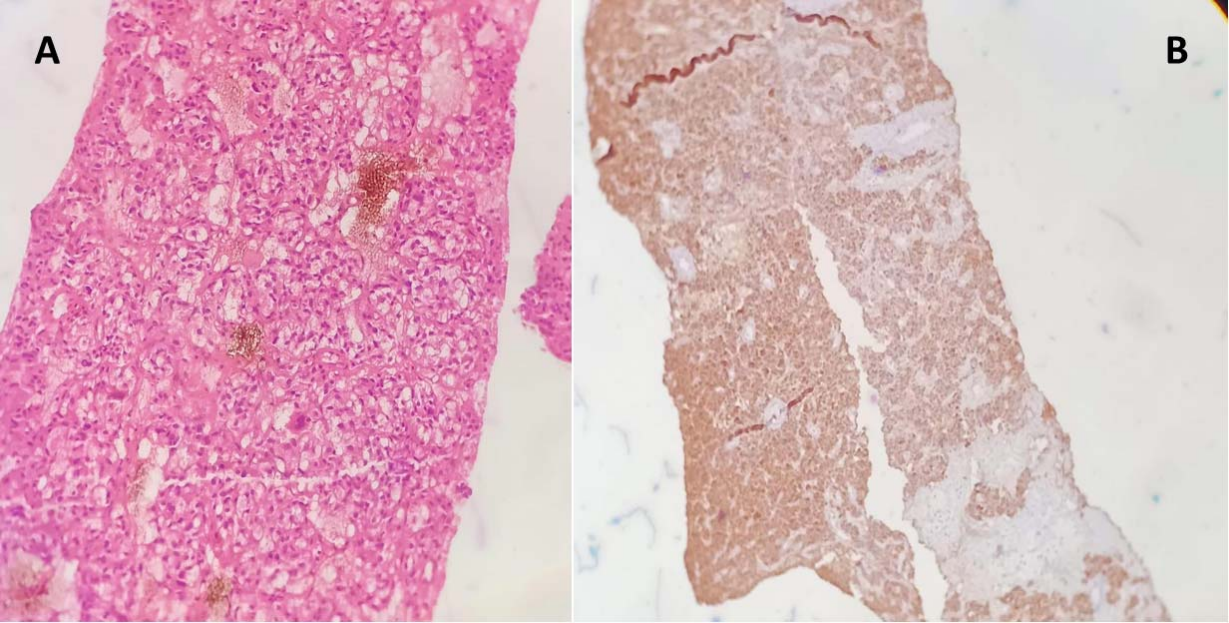
(A) Histopathology demonstrates nests, packets, and sheets of polygonal cells with pleomorphic, hyperchromatic round to ovoid nuclei and intranuclear inclusions 1-2 mitosis per 30 hpf. (B) Tumor cells stained positive for chromogranin.

**Figure 5.**
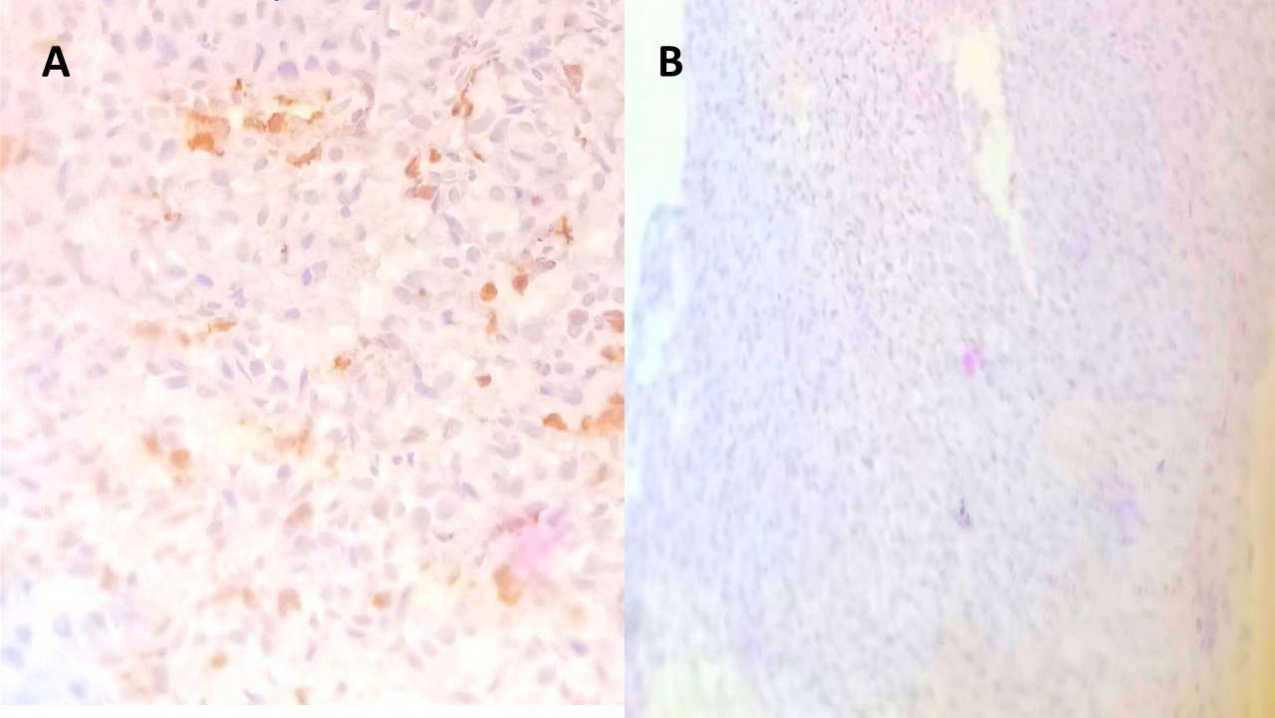
(A) S100 was positive in rare cells. (B) Cytokeratin was negative in tumor cells.

Using an 18-gauge needle, an ultrasound-guided tru-cut biopsy of the right suprarenal mass was obtained. Histopathology demonstrated nests and sheets of cells with hyperchromatic nuclei. All tumor cells were positive for chromogranin (Figure 4). S100 was positive in a few tumor cells and cytokeratin was negative in all tumor cells, confirming pheochromocytoma (Figure 5).

A plan for multidisciplinary team care was devised, and the patient was offered debulking surgery that he declined. The patient agreed to palliative medical therapy with carvedilol, prazosin, and analgesics. The patient was discharged from the hospital, and palliation continued until the patient died 3 months later.

## DISCUSSION

A study from Pakistan reported that the most common symptoms for which patients with pheochromocytoma seek medical attention are abdominal pain, hypertension, and headache, in that order.^14^ Although our patient had no known history of hypertension, vitals monitoring during his 2-week hospital stay revealed persistent hypertension and tachycardia. While gastrointestinal symptoms (eg, abdominal discomfort, nausea, and vomiting) are frequently associated with pheochromocytomas, the occurrence of hematemesis and melena in our patient was an unusual manifestation.

Pheochromocytomas presenting with gastrointestinal hemorrhage have rarely been reported.^15^ Ugur et al reported the case of a patient with esophageal varices and an underlying pheochromocytoma without hepatic metastasis.^16^ The patient's gastrointestinal bleed did not recur after resection of the tumor. Some authors have proposed that the catecholamine excess from these tumors may lead to vascular malformations such as vascular aneurysms and ectasias that are prone to rupture.^16,17^ We, however, believe that our patient developed lower esophageal varices because of severe portal hypertension that resulted from extensive hepatic metastasis and thrombotic obstruction of the IVC.

Metastasis to the liver, as seen in our patient, is a typical manifestation of malignant pheochromocytomas. However, invasion of the IVC with infiltration into the right ventricle resulting in tricuspid valve malfunction is a rare finding. Intravascular extension of the tumor thrombus into the IVC has been rarely reported,^12,13^ and growth of the tumor into the heart is even rarer.^18^ The occurrence of IVC thrombosis with pheochromocytomas is thought to be caused by various mechanisms including compression of the IVC by the tumor mass resulting in blood stasis, a hypercoagulable state associated with malignancy, or intravascular proliferation of the tumor mass itself.^12^

Patients diagnosed with malignant pheochromocytomas have a median survival of 7.2 years.^19^ One fatal complication is hypertensive crisis caused by the release of large amounts of catecholamines in the blood. Treatment involves surgical removal of the primary tumor. In malignant cases, different therapeutic techniques are employed to reduce tumor mass in metastatic locations, including hepatectomy for hepatic metastasis,^20^ tumor embolization, and use of radionuclide agents. For cases that involve the IVC, a thoracolaparotomy has been performed for partial resection of the IVC with successful results.^18^ In all cases, preoperative medical management includes the control of tachycardia and hypertension by administration of alpha adrenergic and beta blocker therapy. All patients require long-term follow-up.

## CONCLUSION

Although rare, malignant pheochromocytomas may present with upper gastrointestinal bleeding. Pheochromocytomas are highly aggressive tumors that can invade the IVC and extend into the heart.

## Notes

*This article meets the Accreditation Council for Graduate Medical Education and the American Board of Medical Specialties Maintenance of Certification competencies for Patient Care and Medical Knowledge.*
